# NOTCH3 and CADASIL syndrome: a genetic and structural overview

**DOI:** 10.14806/ej.24.0.921

**Published:** 2019-05-22

**Authors:** Eleni Papakonstantinou, Flora Bacopoulou, Dimitrios Brouzas, Vasileios Megalooikonomou, Domenica D’Elia, Erik Bongcam-Rudloff, Dimitrios Vlachakis

**Affiliations:** 1 Laboratory of Genetics, Department of Biotechnology, School of Food, Biotechnology and Development, Agricultural University of Athens, Athens, Greece; 2 Lab of Molecular Endocrinology, Center of Clinical, Experimental Surgery and Translational Research, Biomedical Research Foundation of the Academy of Athens, Athens, Greece; 3 Center for Adolescent Medicine and UNESCO Chair on Adolescent Health Care, First Department of Pediatrics, Medical School, National and Kapodistrian University of Athens, Aghia Sophia Children’s Hospital, Athens, Greece; 4 1st Department of Ophthalmology, National and Kapodistrian University of Athens, Athens, Greece; 5 Computer Engineering and Informatics Department, School of Engineering, University of Patras, Patras, Greece; 6 CNR Institute for Biomedical Technologies, Bari, Italy; 7 SLU-Global Bioinformatics Centre, Department of Animal Breeding and Genetics Science, University of Agricultural Sciences, Uppsala, Sweden; 8 Department of Informatics, Faculty of Natural and Mathematical Sciences, King’s College London, London, United Kingdom

## Abstract

CADASIL syndrome is a rare disease that belongs to a group of disorders called leukodystrophies. It is well established that NOTCH3 gene on chromosome 19 is primarily responsible for the development of the CADASIL syndrome. Herein, an attempt is made to shed light on the actual molecular mechanism underlying CADASIL syndrome, through insights extracted from comprehensive evolutionary studies and in silico modelling on Notch 3 protein. In particular, we suggest the use of optical coherence tomography angiography for the detection of early signs of small vessel diseases, which are the major precursors to a repertoire of neurodegenerative conditions, including CADASIL.

## Introduction

The CADASIL (Cerebral Autosomal Dominant Arteriopathy with Subcortical Infarcts and Leukoencephalopathy) syndrome is a hereditary dominant rare disease caused by NOTCH3 gene mutations, affecting adults over the middle-age and leading to dementia and disability.

At systemic level CASADIL syndrome is characterised by a series of damages at the central nervous system produced by recurrent ischemic strokes accompanied by diffuse white matter lesions and subcortical infarcts. Among all known rare diseases, CASADIL is one of the most common form of hereditary stroke disorder that primarily affects small blood vessels in the white matter of the brain (Guruharsha, 2012), and is distinguished from other vascular diseases by the characteristic accumulation of granular osmiophilic material in brain vasculature (Tikka *et al.,* 2009).

**Figure 1.**
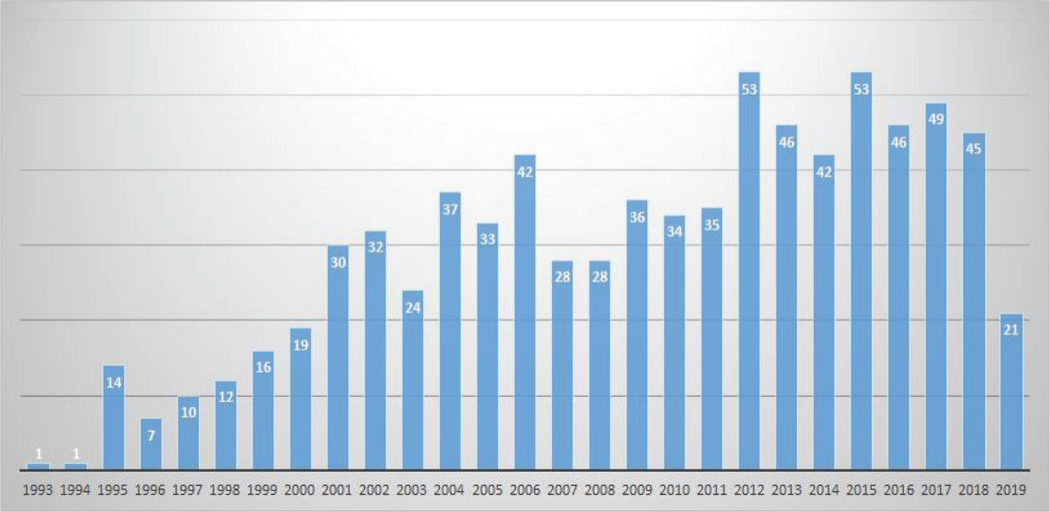
PubMed research results for articles published on the CASADIL syndrome. Search term “Cerebral Autosomal Dominant Arteriopathy with Subcortical Infarcts AND Leukoencephalopathy” in the [Title/Abstract] fields.

The most recent updating (Last Update: March 14, 2019) from GeneReviews^1^, an international point-of-care resource for clinicians, only provides some recommendations to supportive treatment of strokes and to alleviate/limit the extent of symptoms such as frequent migraine, mood disturbance, apathy and the progressive cognitive decline to dementia. The efficacy of prevention of primary manifestations such as stroke/TIA has not still been proven, and surveillance is demanded to clinician’s specialists. The instruments available for diagnosis are the genetic analysis and magnetic resonance imaging (MRI) evaluation. As for any rare disease, the number of studies is still too small (Figure 1), whereas to elucidate the molecular mechanisms underlining CASADIL syndrome is crucial to provide people affected by this disease with a hope for effective therapy.

First studies identifying in mutations of the NOTCH3 gene the genetic origin of the CASADIL syndrome were published by (Joutel *et al.,* 1996), after a previous study that mapped, through genetic linkage analysis in two unrelated families, the disease locus to chromosome 19q12 (Tournier-Lasserve *et al.,* 1993). Since then, the majority of efforts have been focused on the study of NOTCH3 and more than 200 mutations have been reported. Some of these mutations give out a phenotype while others remain silent. Extensive analysis for grouping, organising and mapping these mutations is essential for a straightforward linkage of genotype-phenotype.

## NOTCH3 mutations in CASADIL syndrome

Notch3 is a large type I transmembrane receptor, mainly expressed in vascular smooth muscle cells and pericytes close to the local blood vessels. It has been reported that if Notch switches off becoming inactive, epidermal precursors kick in that convert normal cells to neuroblasts (Artavanis-Tsakonas and Muskavitch, 2010; Louvi and Artavanis-Tsakonas, 2012; Poulson, 1937; Vlachakis *et al.,* 2014). Neuroblasts differentiate and produce embryos which are characterised by nervous system hypertrophy and epidermal structure deficiencies. Accumulation and deposition of Notch3 extracellular domain within vessel walls is a key pathological feature in CADASIL patients and is believed to be responsible for the formation of granular osmiophilic material (GOM) on the surface of vascular smooth muscle cells and pericytes. For this reason, GOM has diagnostic value for CASADIL syndrome when observed in ultrastructural analyses of skin biopsies.

So far, a series of Notch3 pathogenic mutations affecting the number of cysteine residues in the extracellular domain of the receptor and causing protein misfolding and receptor aggregation have been identified. Some of these mutations that lead to the loss or gain of a cysteine residue in 1 of the 34 epidermal growth factor-like repeat (EGFr) domains of the protein. These mutations are highly distinctive for the CASADIL syndrome. Several studies have been carried out to determine their penetrance and frequency. A recent work reports that NOTCH3 EGFr 1–6 pathogenic variants are much more frequent in CASADIL patients than EGFr 7–34 pathogenic variants, which instead predominate in the population. NOTCH3 EGFr 1–6 pathogenic variants are also associated with a more severe phenotype, are characterized by a 12-year earlier onset of stroke, and by a lower survival of CASADIL patients compared with EGFr 7–34 pathogenic variants (Rutten *et al.,* 2019). However, also cysteine-sparing NOTCH3 mutations have recently been identified and do not follow the characteristic pathology and pattern of the disease. Some of these cysteine-sparing NOTCH missense mutations also cause GOM whereas some others do not (Muiño *et al.,* 2017). The pathogenic role of cysteine-sparing NOTCH3 mutations in patients with typical clinical CADASIL syndrome is still unknown.

## Linking known Notch3 mutations to structure

**Figure 2.**
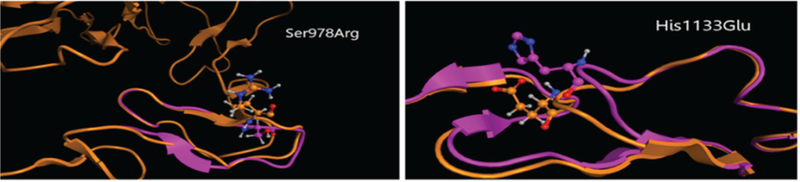
3D molecular modelling studies on point mutations of Notch 3 indicates a significant loss of 3D structure, thus resulting in a partially unfolded protein with compromised functionality. The study was performed in the Molecular Operating Environment (MOE 2011.10). Montreal, Quebec, Canada: Chemical Computing Group ULC^2^

The specific aim of this study is to provide insights into the structural properties of the Notch3 protein which promote CADASIL. This can be achieved by analyzing the 3D structural properties of the Notch3 protein. Through our preliminary genetic and proteomic analysis of the Notch3 protein (Dr Baumann group), we are aware of a series of point mutations of the Notch3 protein which lead to loss of structure and therefore promote CADASIL (Figure 2) (Polychronidou *et al.,* 2015).

The link between the CADASIL manifestation and the Notch3 3D structure is mainly based on the partially known X-ray crystallography Notch3 3D structure. Correlation of the mutant Notch3 proteins to the wild type model has provided us with all the vital structural information concerning the extent and nature of Notch3 structural loss. The structurally and conformationally essential sites on the Notch3 structure have been identified so this information could lead to future in silico and in vitro experiments to be conducted to analyse their effect and role in CADASIL (Ioannidou *et al.,* 2013).

## Evolutionary study of Notch 3

During this first phase of the phylogenetic analysis, motif construction and codon usage have been conducted (Vlachakis *et al.,* 2014). The Notch3 protein and nucleotide sequences have been retrieved from the literature and the publicly available genomic databases. To determine the phylogenetic status of the Notch3 protein, detailed and comprehensive phylogenetic analysis of protein sequences has been conducted. Subsequently, sequence motifs were excised from the alignments. It has been demonstrated that different genomes have their characteristic patterns of codon usage. Based on the above, we have investigated the variations in codon usage among the different NOTCH3 genomes. We have evaluated if there are any characteristic patterns of codon usage and how these patterns are related to the CADASIL disease. Towards this direction, we have combined the genes from all NOTCH3 genomes and calculated the relative codon usage for each genome and each gene per genome by using the Codon Adaptation Index (CAI) (Sharp and Li, 1987).

## Genetic study of NOTCH3

Statistical analysis was employed to explore all sequence variations and Single Nucleotide Polymorphisms (SNP) for NOTCH3 (Polychronidou *et al.,* 2015). Sequence variation was further analysed by exploring any relationship or patterns among SNPs within the species. To identify the individual nucleotide positions which most contribute to the NOTCH3 differentiation, a list of all informative phylogenetic sites from the codon-aligned multiple genome alignment was assembled. Then, a consensus sequence was created by the “majority rule” Subsequently, a Bayesian partition model was employed to identify groups of more than two SNPs in the data exhibiting similar behaviour, estimating their optimum number and distributional properties in the genomes considered. By using the latter approach, we aimed to enhance our understanding of the dependencies between the various motifs examined for all the populations and consequently their involvement in the CADASIL. Past studies on NOTCH3 genetics have employed standard statistical techniques or methodologies which allow exploring genetic data and identify natural groupings of most similar sequences; however, here we suggest a likelihood-based model approach where whole sequence interactions are considered without restricting our search to pair-wise or threshold restricted groups of SNPs similarities.

## Structural study of Notch 3

The next step included the structural analysis task of the multiple known mutations of Notch3 (Vlachakis *et al.,* 2014). Notch3 structural features were explored, parameterised and prepared as input information for our pipeline. We induced the various known mutations of Notch3 on the wild type 3D structure of Notch3 which has been made available by X-ray crystallography experiments. Then, using energy minimisation and molecular dynamics simulations, the structural significance of the induced mutations was evaluated in silico. Investigation of the resulting molecular conformations of the mutants shed light to the structural properties of the Notch3 3D organisation which lead to the activation or deactivation of the Notch3 protein and eventually to CADASIL disease.

Moreover, we already know that the 3D structural arrangement of Notch 3 protein is very similar to that of Fibrillin. A series of many Fibrillin mutations which lead to the Marfan syndrome have been published in literature so far. We mimicked those Fibrillins mutations to the Notch3 protein. Therefore, mapping all the known mutations on the Notch 3 structure and then subsequently projecting them on the 3D fold of Fibrillin have yielded invaluable results since the latter’s structure and properties have been well studied and reported in tha literature.

Next, we performed homology modelling of the various mutant constructs of Notch3 protein. The homology modelling algorithm performed an initial partial geometry alignment for the sequence of the template proteins with the Notch3 sequence. All available substrates and ligands of Notch3 were added to the model to establish the specific interaction patterns and “key” residues involved in the ligand – Notch3 interaction. Similarities or complementarities between the surfaces of Notch3 and other X-ray determined structures have provided crucial insights on structural conservation among these proteins that relate to their mechanism of action and function.

High throughput virtual screening techniques were used to screen all available substances with orphan drug designation from EMA. To date, the EMA orphan drug database contains 1727 entries. Molecular dynamics and molecular docking simulations were applied to evaluate the association and binding efficacy of each one of the orphan drugs to the wild type Notch3 protein and the models of Notch3 mutants. Using molecular dynamics, we were able to monitor the motion of atoms and molecules in a computerised biological system.

## Notch 3 structure - phenotype map

Finally, a 3D interactive Notch3 specific prediction structure -phenotype map was established (Vlachakis *et al.,* 2014). Each amino acid position on the Notch3 protein was linked to the phenotype it produces, so that the phenotype of new/future mutations may be predicted in silico. This final part of the study aims to combine all the information gathered from all previous steps in an easy to comprehend graphic representation of Notch3 protein. This way it is possible firstly to get updated with the current research on NOTCH3 and CADASIL, as well as to predict the effect that a new mutation may have, based on the position of the mutation on the 3D structure and the physicochemical alteration which the mutation induces. Emphasis was paid to the Cysteine residue mutations which usually lead to unpaired Cysteine residues and promote the CADASIL disease. Even though CADASIL is a rare disease, its linkage to NOTCH3 makes it very interesting from a basic research point of view. We believe that the establishment of an interactive map of Notch3 genotype/phenotype on the 3D structure of Notch3 can bring the scientific community up to speed with current developments in this exciting field.

## CADASIL and optical coherence tomography angiography

Abnormalities on brain imaging, usually MRI, often exist in CASADIL patients long before symptoms occur, and seem to have a faster rate of progression than other clinical outcome measures, such as cognitive impairment or stroke incidence. For this reason MRI can be used as surrogate biomarkers of the disease sensitive to change in time. Using digital imaging of the retinal area has been growing increasingly common, and can be used for the analysis of vascular topography, including the width of retinal microvessels (Patton *et al.,* 2005). Retinal vasculature can be visualised in vivo and photographed in 2D. Hence, it has great potential of being used as an index, given the anatomical correlation between both the macrovascular and the microvascular blood supply, to the retina and the brain. Fractal analysis has been used to measure the complexity, or density, of the retinal vessel branching, expressed by the mean fractal dimension (mean-D) value, based on the hypothesis that reduced mean-D in CADASIL patients reflects the cerebral microvascular changes associated with the disease progression (Cavallari *et al.,* 2011).

Similarly, optical coherence tomography angiography (OCT-A), a new non-invasive imaging technique which generates volumetric angiography images of the retina, may be used as a surrogate outcome marker, though less validated compared with MRI markers (de Carlo *et al.,* 2015). Using OCT-A, it is possible to detect changes in the retinal microcirculation and generate a blood flow map. A recent work by (Nelis *et al.,* 2018), reports about a study in which they found a significant decrease in macular vessel density in the deep retinal plexus in CADASIL patients compared to healthy control, therefore supporting the use of this technology to detect the disease in asymptomatic individuals and to monitor the progression of the disease in patients.

## Conclusions

Based on our already demonstrated results (via exhaustive molecular dynamics simulations) it was found that non-Cys mutations trigger significant loss of structure in the Notch3 protein, compared to the wild type. To identify the underlying mechanism of Notch3 role and implications in cell signal transduction, an investigation was performed to the nature, extent, physicochemical and structural significance of the mutant species. Preliminary in silico studies revealed a rather complex molecular mechanism on the structural level. Even though there are mainly point mutations, the effect of each one of them on the three-dimensional structure of the Notch3 protein is significant. However, in some cases, although local rearrangements in structure are observed, the overall 3D structural conformation of Notch3 remains quite unchanged. Finally, the structural similarity of Notch3 and Fibrillin was explored to transfer knowledge regarding structural characteristics and ligands from the well-studied field of Fibrillin to the Notch3 research domain.

Currently, there is none therapeutic treatment available for CADASIL and thereof no drug which can act specifically on the Notch3 protein receptor. Medical practitioners prescribe aspirin, dipyridamole, or clopidogrel, or a combination of these, which are found to limit the symptoms of the disease and to relatively slow it down. Given that all conventional attempts have failed in identifying a disease-modifying treatment, an extensive in silico analysis of the Notch3 mutations and of the resulting angiogenic plasticity of the CADASIL phenotype on small vessels, could potentially lead to a radical early detection pipeline. The latter coupled by recent breakthroughs in OCT-A technology, image analysis and computational biology are steadily gaining ground in neurodegenerative disease treatments under the emerging prism of preventive and precision medicine.

## Key Points

- The CADASIL syndrome is a hereditary dominant rare disease, caused by NOTCH3 gene mutations.
- A series of Notch 3 pathogenic mutations have been identified to cause protein misfolding and receptor aggregation.
- Accumulation and deposition of Notch 3 extracellular domain within vessel walls is a key pathological feature in CADASIL patients.
- Optical coherence tomography angiography (OCT-A) represents the most recent tool in ophthalmic imaging for the detection of early signs of small vessel diseases.
- OCT-A is a very effective and non-invasive tool for the investigation of CASADIL in asymptomatic individuals and to monitor the progression of the disease in patients.

## Footnotes

1 https://www.ncbi.nlm.nih.gov/books/NBK1500/

2 http://www.chemcomp.com
